# Genome-wide mapping of TnrA-binding sites provides new insights into the TnrA regulon in *Bacillus subtilis*

**DOI:** 10.1002/mbo3.249

**Published:** 2015-03-08

**Authors:** Nicolas Mirouze, Elena Bidnenko, Philippe Noirot, Sandrine Auger

**Affiliations:** 1 UMR1319 Micalis, INRAF-78352, Jouy-en-Josas, France; 2 UMR Micalis, AgroParisTechF-78352, Jouy-en-Josas, France

**Keywords:** *Bacillus subtilis*, ChIP-on-chip, nitrogen metabolism, oxidative stress, TnrA regulator

## Abstract

Under nitrogen limitation conditions, *Bacillus subtilis* induces a sophisticated network of adaptation responses. More precisely, the *B. subtilis* TnrA regulator represses or activates directly or indirectly the expression of a hundred genes in response to nitrogen availability. The global TnrA regulon have already been identified among which some directly TnrA-regulated genes have been characterized. However, a genome-wide mapping of in vivo TnrA-binding sites was still needed to clearly define the set of genes directly regulated by TnrA. Using chromatin immunoprecipitation coupled with hybridization to DNA tiling arrays (ChIP-on-chip), we now provide in vivo evidence that TnrA reproducibly binds to 42 regions on the chromosome. Further analysis with real-time in vivo transcriptional profiling, combined with results from previous reports, allowed us to define the TnrA primary regulon. We identified 35 promoter regions fulfilling three criteria necessary to be part of this primary regulon: (i) TnrA binding in ChIP-on-chip experiments and/or in previous in vitro studies; (ii) the presence of a TnrA box; (iii) TnrA-dependent expression regulation. In addition, the TnrA primary regulon delimitation allowed us to improve the TnrA box consensus. Finally, our results reveal new interconnections between the nitrogen regulatory network and other cellular processes.

## Introduction

In bacteria, nitrogen is present in nearly all macromolecules such as proteins, nucleic acids, and peptidoglycan. To provide an optimal supply of this macronutrient, prokaryotes have developed transport and assimilation systems for a variety of nitrogen sources as well as sophisticated control mechanisms to ensure energy-efficient uptake and assimilation. This regulatory network allows an adequate response to situations of nitrogen limitation.

In the Gram-positive bacterium *Bacillus subtilis*, ammonium assimilation occurs via the glutamine synthetase-glutamate synthase (GS-GOGAT) pathway to generate glutamate, the precursor for amino acids and nucleotides biosynthesis (Dean and Aronson 1980). *Bacillus subtilis* faces nitrogen- limiting conditions when growing with glutamate as the sole nitrogen source (Atkinson and Fisher 1991) while glutamine is the preferred nitrogen source followed by arginine and ammonium (Hu et al. 1999; Fisher and Debarbouille 2002).

Two transcription factors, TnrA and GlnR, and one enzyme, the GS, are the major players in the *B. subtilis* nitrogen regulatory network (Schreier et al. 1989; Wray et al. 1996; Fisher 1999). TnrA and GlnR both control the expression of nitrogen-regulated genes with partial overlap of their respective regulon. In addition, they cross-regulate each other's expression. Under nitrogen-limited conditions, TnrA can act both as activator and repressor (Wray et al. 2001). It upregulates expression of genes encoding ammonium transport (*amtB-glnK* originally named *nrgAB*) (Wray et al. 1994), *γ*-aminobutyrate permease (*gabP*) (Ferson et al. 1996), nitrate assimilation (*nasA* and *nasB* operons) (Nakano et al. 1995), nitrate assimilatory enzymes (*nasDEF*) (Nakano et al. 1998), glutamine uptake (*glnQHMP*) (Yoshida et al. 2003), asparginase (*ansZ*) (Fisher and Wray 2002), guanine deaminase (*guaD* originally named *yknA*) (Nygaard et al. 2000), purine catabolism (*pucJKLM* operon) (Schultz et al. 2001), oligopeptide uptake (*opp* operon) (Yoshida et al. 2003), *kipI* (Wang et al. 1997), *ykzB-ykoL* (Robichon et al. 2000), *ywrD* (Yoshida et al. 2003), and its own gene *tnrA* (Fisher 1999; Robichon et al. 2000). TnrA also exerts activating effect on expression of the aconitase gene, *citB*, but the mechanism of regulation remains still unclear (Blencke et al. 2006). On the other hand, TnrA represses expression of *glnRA* (GS) (Brown and Sonenshein 1996; Wray et al. 1996; Zalieckas et al. 2006), *gltAB* (glutamate synthetase) (Belitsky et al. 2000), *ilv-leu* (synthesis of branched-chain amino acids) (Tojo et al. 2004), *pel* (pectate lyase C) (Yoshida et al. 2003), *degU* (DegS-DegU two-component system) (Yasumura et al. 2008), *alsT*, *ywdIJK*, *yycCB*, *yttA*, *yxkC*, *ywlFG*, and *yodF* (Yoshida et al. 2003).

Glutamine acts as the metabolic signal for nitrogen availability. The form of GS that is feedback inhibited by excess glutamine directly interacts with and sequesters TnrA, thus blocking its DNA-binding activity (Wray et al. 2001). TnrA loses control over its regulon and transcriptional control is taken over by GlnR. GlnR acts as a repressor of *glnRA*, *ureABC*, and *tnrA* expression (Schreier et al. 1989; Brown and Sonenshein 1996; Wray et al. 1997; Fisher 1999). Moreover, CodY, a third regulatory protein, responds to the total nutritional state of the cell by controlling genes involved in nitrogen utilization as well as carbon metabolism, competence, sporulation, and motility (Ratnayake-Lecamwasam et al. 2001; Molle et al. 2003).

Previous transcriptomic analysis of *B. subtilis* grown under nitrogen-limiting or excess growth conditions revealed the changes of hundred of genes. The expression of these genes could be directly or indirectly controlled by TnrA (Jarmer et al. 2002; Ye et al. 2009). Seventeen TnrA targets were detected by a combination of DNA microarray hybridization, a genome-wide search for TnrA boxes, and gel retardation assays (Yoshida et al. 2003). The TnrA box consensus delimited in this study is a 17-bp interrupted, inverted repeat sequence, TGTNANAWWWTNTNACA.

Although some TnrA-regulated genes have already been well characterized, a global identification of the genes directly under TnrA control was still missing. Here, we used chromatin immunoprecipitation of TnrA-DNA complexes coupled with hybridization of DNA to tiled oligonucleotides arrays (ChIP-on-chip) to identify the TnrA DNA-binding sites, in vivo, at the genome scale. We provide evidence that TnrA binds reproducibly to 42 regions on the chromosome. In combination with real time in vivo transcriptional profiling using firefly luciferase, our data allowed us to define the TnrA primary regulon, which is now composed of 35 promoter regions. Thanks to this restricted list of genes directly regulated by TnrA, we proposed an improved TnrA box consensus. In addition, we characterized the TnrA secondary regulon, which is composed of promoter regions harboring a TnrA box and bound by TnrA in vivo. However, the growth conditions revealing a TnrA-dependent regulation for this second category of genes remain still unknown. Finally, our results highlight connections between the nitrogen metabolism and other regulatory networks.

## Experimental Procedures

### Bacterial strains and growth conditions

The *B. subtilis* strains used in this work are listed in Table1. *Bacillus subtilis* cells were grown in Luria–Bertani (LB) medium or in minimal medium containing 62 mmol/L K_2_HPO_4_, 44 mmol/L KH_2_PO_4_, 17 mmol/L trisodium citrate, 11 mmol/L K_2_SO_4_, 0.6% glycerol, 1 mmol/L MgSO_4_, 1 mmol/L CaCl_2_, 100 *μ*mol/L FeCl_3_ citrate, 112 *μ*mol/L ZnCl_2_, 5 *μ*mol/L MnCl_2_, 2.5 *μ*mol/L CuCl_2_, and 0.3% glutamate. Glutamine was added at the final concentration of 0.3% when needed. *Escherichia coli* cells were grown in LB medium. Antibiotics were added at the following concentrations when required: 100 *μ*g ampicillin mL^−1^; 5 *μ*g chloramphenicol mL^−1^; 60 *μ*g spectinomycin mL^−1^. Solid media were prepared by the addition of 20 g Agar noble L^−1^ (Difco, New Jersey, USA). Standard procedures were used to transform *E. coli* (Sambrook et al. 1989) and *B. subtilis* (Kunst and Rapoport 1995).

**Table 1:** *Bacillus subtilis* strains used in this work

Strain	Genotype	Source
BSB1	*trp*^*+*^	Nicolas et al. (2012)
Bas055	*tnrA*::*tnrA-spa erm*	This work
BSB53	Δ*tnrA*::*spc*	This study
BLUC45	P*nasB′-luc cat*	This study
BLUC46	P*nasB′-luc cat* Δ*tnrA*::*spc*	This study
BLUC49	P*hom′-luc cat*	This study
BLUC50	P*hom′-luc cat* Δ*tnrA*::*spc*	This study
BLUC51	P*yuiA′-luc cat*	This study
BLUC52	P*yuiA′-luc cat* Δ*tnrA*::*spc*	This study
BLUC59	P*yfiR′-luc cat*	This study
BLUC60	P*yfiR′-luc cat* Δ*tnrA*::*spc*	This study
BLUC61	P*pycA′-luc cat*	This study
BLUC62	P*pycA′-luc cat* Δ*tnrA*::*spc*	This study
BLUC65	P*appD′-luc cat*	This study
BLUC66	P*appD′-luc cat* Δ*tnrA*::*spc*	This study
BLUC67	P*tdh′-luc cat*	This study
BLUC68	P*tdh′-luc cat* Δ*tnrA*::*spc*	This study
BLUC75	P*pucR′-luc cat*	This study
BLUC76	P*pucR′-luc cat* Δ*tnrA*::*spc*	This study
BLUC77	P*dtpT′-luc cat*	This study
BLUC78	P*dtpT′-luc cat* Δ*tnrA*::*spc*	This study
BLUC79	P*yrbD′-luc cat*	This study
BLUC80	P*yrbD′-luc cat* Δ*tnrA*::*spc*	This study
BLUC81	P*ysnD′-luc cat*	This study
BLUC82	P*ysnD′-luc cat* Δ*tnrA*::*spc*	This study
BLUC83	P*yvgT′-luc cat*	This study
BLUC84	P*yvgT′-luc cat* Δ*tnrA*::*spc*	This study
BLUC85	P*alsT′-luc cat*	This study
BLUC86	P*alsT′-luc cat* Δ*tnrA*::*spc*	This study
BLUC93	P*alsT′-luc cat tnrA*::*tnrA*-*spa erm*	This study
BLUC97	P*yclN′-luc cat*	This study
BLUC98	P*yclN′-luc cat* Δ*tnrA*::*spc*	This study
BLUC121	P*pucI′-luc cat*	This study
BLUC122	P*pucI′-luc cat* Δ*tnrA*::*spc*	This study
BLUC125	P*pucA′-luc cat*	This study
BLUC126	P*pucA′-luc cat* Δ*tnrA*::*spc*	This study

### DNA manipulations

DNA manipulations and cloning procedures were performed as described elsewhere (Sambrook et al. 1989). Restriction enzymes, *Pfu* DNA polymerase and phage T4 DNA ligase were used as recommended by the manufacturer (Biolabs, Ipswich, MA, USA). DNA fragments were purified from agarose gels using the QIAquick kit (Qiagen, Hilden, Germany).

### Construction of a tnrA::tnrA-spa strain

A *B. subtilis* strain expressing a C-terminal SPA-tagged TnrA protein (hereafter TnrA^SPA^) was constructed by chromosomal integration of a translational fusion between the *tnrA* coding sequence and the sequential peptide affinity (SPA) tag sequence (Zeghouf et al. 2004; Butland et al. 2005), resulting in the Bas055 strain expressing TnrA^SPA^ under the control of its native promoter as unique source of TnrA. In this purpose, the *tnrA* coding sequence (from nucleotide +6 to +324 relative to the translational start site) was amplified by PCR with oligonucleotides creating an *Acc*651 restriction site at the 5′ end and a *Nco*I restriction site at the 3′ end of the fragment. The PCR product was cloned into plasmid pMUTIN-SPA subsequent to digestion with *Acc*651 and *Nco*I (Lecointe et al. 2007). The resulting plasmid was used to transform *B. subtilis* and to select for erythromycin resistance. Integration was confirmed by PCR and verified by DNA sequencing.

### Construction of ΔtnrA deletion

The *tnrA* mutant BSB53 was constructed by homologous replacement of the *tnrA* coding sequence with the spectinomycin-resistant gene *spc* using a joining PCR technique (Wach 1996). The *spc* gene was first amplified. The region upstream of the *tnrA* gene (nucleotides 1396411 to 1397471) was amplified by PCR with a 24-bp *spc* fragment at its 3′ end. The region downstream of *tnrA* (nucleotides 1397671 to 1398742) was amplified with a 24-bp *spc* fragment at its 5′ end. The three DNA fragments were combined and then a PCR reaction was performed with the two external oligonucleotides. The final product, corresponding to the two regions flanking *tnrA* with the inserted *spc* cassette in between, was purified from a gel and used to transform *B. subtilis*. Integration and deletion were confirmed by PCR and verified by DNA sequencing.

### Construction of luciferase promoter fusion strains

The pUC18cm-luc plasmid was first amplified with primers resulting in a 5400-bp linear product (Table S3). A 1-kb fragment ending with the initiating codon of the gene of interest, and containing the promoter, was amplified by PCR from the *B. subtilis* chromosome. The used primers contained extremities matching with the pUC18cm-luc plasmid (Table S3). The linear plasmid and the 1-kb fragment were mixed with the enzymes mix according to the assembly Gibson's procedure (Gibson et al. 2009) to obtain the integration of the fragment into the plasmid. Each resulting plasmid, pUC18cm-promoter::luc, which cannot replicate autonomously in *B. subtilis*, was used to transform *B. subtilis* where it integrated, by single crossover. This event reconstructs the “normal” regulatory region in front of the fusion and a complete copy of the gene of interest, downstream of the fusion.

### Luciferase assay

For the detection of luciferase activity, strains were first grown in LB medium to an optical density at 600 nm (OD_600_) of 2. Cells were then centrifuged and resuspended in fresh minimal medium, adjusting all the cultures to an OD_600_ of 2. These precultures were then diluted 20-fold in fresh minimal medium and 200 *μ*L was distributed in each of two wells in a 96-well black plate (Corning, New York, USA). Ten microliters of luciferin was added to each well to reach a final concentration of 1.5 mg/mL (4.7 mmol/L). The cultures were incubated at 37°C with agitation in a PerkinElmer Envision 2104 Multilabel Reader (Perkin Elmer, Waltham, MA, USA) equipped with an enhanced sensitivity photomultiplier for luminometry. The temperature of the clear plastic lid was maintained at 38°C to avoid condensation. Relative Luminescence Unit (RLU) and OD_600_ were measured at 5-min intervals.

### Genome-wide determination of the TnrA-binding sites by ChIP-on-chip

Chromatin Immnunoprecipitation assays were performed to measure the chromosome-wide DNA-binding profiles of TnrA, as described previously (Nicolas et al. 2012). Briefly, strain Bas055 was cultivated at 37°C until an OD_600_ of 0.6 in LB or minimal medium supplemented with 0.5 mmol/L IPTG isopropyl β-D-1-thiogalactopyranoside and 1 *μ*g erythromycin mL^−1^. Cells were treated with formaldehyde, cellular DNA was extracted and sonicated, and an antibody against the FLAG epitope tag (DKYDDDK) was used to preferentially purify the DNA regions specifically cross-linked to TnrA^SPA^. The immunoprecipitated DNA (IP) and the control whole cell DNA extract (WCE) were labeled with Cy3 and Cy5, respectively, and co-hybridized to the *B. subtilis* Roche-NimbleGen tiled microarrays (Rasmussen, 2009).

### Peak sequence extraction and analysis

Identification of peaks corresponding to chromosomal TnrA-binding sites was performed as described in Reppas et al. (2006). IP/WCE ratios (log2) were corrected for dye bias using Loess regression on the MA plot. The signal was smoothed by two rounds of sliding window averaging (29 probes, around 320 bp). Maxima (or minima) were defined as probes for which the smoothed signal is the highest (or lowest, respectively) into the window used for smoothing. Peaks within the same 300-bp window were merged. The peak height was calculated as the log2 ratio difference between the smoothed signal values of the maxima and the adjacent minima. In order to quantify enrichment of TnrA-bound DNA regions, the signal was smoothed and a ChipScore was calculated as described by Buescher et al. (2012). Briefly, this score is based on the distribution of the peak height values and estimates for each peak its relative distance from the median (ChipScore = [height − median]/[upperquartile − median]). Only the regions associated with a peak scoring ≥4.5 [a threshold determined empirically from ChIP–on-chip experiments with the transcription factor CcpA] (Buescher et al. 2012) in at least two experiments were considered as putative TnrA-binding sites in the subsequent analyses.

### Prediction of a TnrA box consensus

For the prediction of a TnrA box consensus sequence from our data, we used the MEME (Multiple Em for Motif Elicitation) standard bioinformatic method (Bailey et al. 2006). As query sequences, we used: (i) the 17-bp sequences of TnrA boxes from the previously known primary TnrA regulon (Yoshida et al. 2003); (ii) a set of genomic regions 100 bp centered at each of the TnrA-bound sites identified from ChIP-on-chip.

We used this newly defined TnrA box consensus sequence (Fig. 3B) to search TnrA box motifs among the 17 TnrA-binding sites identified in ChIP-on-chip, which did not harbor a previously predicted TnrA box. The number of mismatches allowed was 8.

## Results

### C-terminally SPA-tagged TnrA is a functional regulator

**Figure 1:**
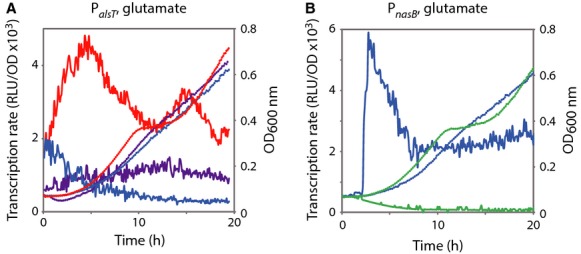
Expression of *alsT* and *nasB* under the control of TnrA. Strains were grown in minimal medium supplemented with glutamate as the sole nitrogen source. Growth was monitored by measuring the optical density at 600 nm in parallel with light emission: blue circles, wild type; red circles, *ΔtnrA*; purple circles, *tnrA*::*tnrA-spa*. (A) Promoter activity of P*alsT′*-*luc* in wild-type (blue line), *ΔtnrA* (red line), and *tnrA*::*tnrA-spa* (purple line) cells. (B) Promoter activity of P*nasB′*-*luc* in wild type (blue line) and *ΔtnrA* (green line) cells. For each strain, one representative curve, out of three independent replicates realized, is shown.

*Bacillus subtilis* chromosome was modified at the *tnrA* locus to express TnrA fused at its C-terminus with the SPA tag (TnrA^SPA^) (see Experimental Procedures). In the resulting *tnrA*::*tnrA*-*spa* strain, expression of the gene encoding the TnrA^SPA^ protein is under the control of its native transcriptional signals. In order to verify if the TnrA^SPA^ protein (used below in the next experiments) was functional, we compared the transcriptional regulation of known TnrA-regulated genes in the presence of TnrA^SPA^ or TnrA^WT^. The promoter region of the *alsT* gene, whose expression is known to be inhibited by TnrA (Yoshida et al. 2003) was fused with the *luc* reporter gene and introduced at the native *alsT* locus in wild-type, *tnrA*::*tnrA*-*spa* and Δ*tnrA*::*spc* strains (Table1). The fusion was integrated by single crossover into the *alsT* promoter region, ensuring that the *luc* reporter was placed under the control of all relevant upstream regulatory sequences and that the wild-type locus was undisturbed. Light emission, which results from the activity of the *luc*-encoded firefly luciferase (Mirouze et al. 2011), was recorded every 5 min during growth in minimal medium with glutamate as sole nitrogen source. The *tnrA*::*tnrA*-*spa* strain growth profile was similar to that of the wild type while the Δ*tnrA*::*spc* mutant presented a slightly affected growth during exponential phase (Fig.1A). Expression of the *alsT* promoter was repressed in the wild-type and *tnrA*::*tnrA*-*spa* strains, whereas it was increased by a fourfold factor in Δ*tnrA* cells (Fig.1A). Thus, TnrA^SPA^ was able to repress *alsT* expression as TnrA^WT^. We concluded from these data that the TnrA^SPA^ fusion protein was functional for transcriptional regulation.

### Genome-wide mapping of TnrA-binding sites

To identify TnrA-binding targets in *B. subtilis* genome, we carried out ChIP-on-chip experiments in two growth conditions. The *tnrA*::*tnrA*-*spa* strain was grown in minimal medium with glutamate as sole nitrogen source and in LB medium to exponential phase. After cross-linking, TnrA-bound DNA was immunoprecipitated using a FLAG-specific antibody. Finally, significantly TnrA-enriched DNA regions were identified as explained in Experimental Procedures.

**Figure 2:**
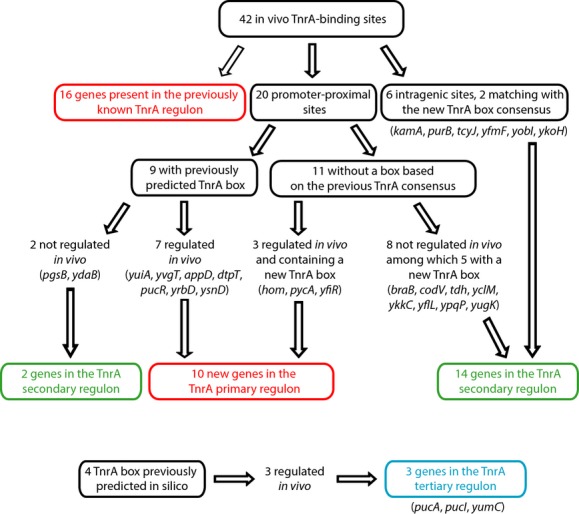
Analysis pipeline of the TnrA-binding sites detected by ChIP-on-chip. The promoter regions associated with TnrA-binding sites were classified in the three groups: TnrA primary (in red), secondary (in green) and tertiary regulon (in blue).

Overall 42 enriched DNA regions ≤200 bp were identified from the ChIP-on-chip signals (Table S1) (Fig.2). To analyze the data, we first focused on the known TnrA-regulated genes (Yoshida et al. 2003; Michna et al. 2014). Sixteen TnrA-binding sites were located in genomic regions known to belong to the global TnrA regulon. These TnrA-bound regions have been shown to regulate 19 transcription units as single binding sites are located in the intergenic region of the divergently transcribed operons *nasA* and *nasB*, *ysnD* and *ilv*, and of the divergent genes *tnrA* and *ykzB* (Table S2). By decreasing the threshold defining a TnrA-binding peak (ChIPScore = 4.0), we did not identify more sites overlapping the so far known TnrA regulon. In total, we retrieved 19 out of 25 well-characterized TnrA-regulated promoters. We did not detect TnrA-binding sites in the promoter regions of *alsT*, *ansZ*, *degU*, *ywlFG*, *ywrD*, and *yxkC* operons.

In addition, 20 additional promoter-proximal TnrA-binding sites were detected less than 300-bp upstream of the start codon (Table S1), suggesting a TnrA-dependent expression and therefore the existence of new candidates in the TnrA regulon. The presence of predicted TnrA boxes in these regions is discussed below.

Finally, six peaks were located within intragenic regions more than 150 base-pairs downstream of the start codon of *kamA*, *purB*, *tcyJ*, *yfmF*, *yobI*, and *ykoH* (Table S1). The location of these sites was intriguing since no TnrA intragenic binding sites have been described so far.

### TnrA-binding sites overlap previous in silico predicted TnrA box

**Figure 3:**
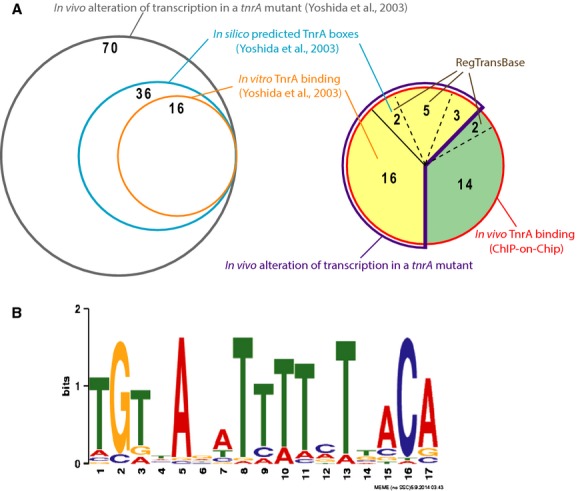
ChIP-on-chip data overlap partly previous studies and allow to generate a new TnrA box consensus. (A) Data obtained in previous studies by transcriptomic and in vitro approaches are presented (left circles) (Yoshida et al. 2003). Forty-two TnrA-binding sites were identified by ChIP-in-chip in this work (red circle, right pie-chart). Twenty-six TnrA-binding sites are associated with promoter regions, whose expression is altered in vivo in a *tnrA* mutant (yellow area). They belong to the TnrA primary regulon. Sixteen TnrA-binding sites are located in inter- as well as intragenic regions but the role of TnrA as a regulator in the associated regions remains still unknown (green area). They belong to the secondary regulon. Comparison of the ChIP-on-chip data with previous studies is indicated. Sixteen TnrA-binding sites are located in promoter regions previously shown to be directly regulated by TnrA (Wray et al. 2001). In total, nine TnrA-binding sites contain a previously predicted TnrA box (Cipriano et al. 2013). (B) Identification of a new consensus of the TnrA-binding motif. The size of the nucleotide at each position correlates woth its relative prevalence in sequences used as training set in the MEME algorithm (Bailey et al. 2006).

To investigate the presence of TnrA boxes within the newly identified 20 promoter-proximal TnrA-binding sites, we compared our data with previous in silico studies available in the RegTransBase database (Fig.3A) (Cipriano et al. 2013) (Leyn et al. 2013). Nine of these enriched regions exhibited TnrA box motifs (Table S1) (Fig.2). These in vivo binding regions were detected less than 100 bp away from putative TnrA boxes, upstream of *appD*, *dtpT*, *ydaB*, *yrbD*, *ysnD*, *yuiA*, *pucH*, *yvgT*, and *pgsB* translational start sites. It is interesting to notice that two TnrA box motifs have been detected in silico in front of the *dtpT*, *glnR*, *ycsF*, and *ysnD* genes correlating with the detection of two TnrA-enriched regions by ChIP-on-chip (Table S1). As putative TnrA boxes actually corresponded to experimentally identified in vivo TnrA-binding sites, the associated genes were assumed to be relevant TnrA-regulated genes candidates.

The 11 other promoter-proximal TnrA-binding sites did not display a significant match to the previously proposed TnrA box consensus (Fig.2). This result suggested that TnrA binding could occur at degenerated TnrA box motifs or that other proteins could be involved in TnrA binding to these DNA sites.

### TnrA-binding sites correlate with transcriptional regulation by TnrA

We then tested the correlation between the presence of TnrA boxes associated with in vivo TnrA-binding and TnrA-dependent expression of the genes found next to them. For this purpose, we constructed transcriptional fusions between the promoter regions containing the TnrA boxes and the luciferase gene in wild-type and Δ*tnrA* cells. Luciferase activity was recorded during exponential growth in minimal medium with glutamate as sole nitrogen source. Two transcriptional fusions containing the *nasB* and *alsT* promoter regions were used as controls of these experiments. As expected in the growth conditions used, expression of P*nasB*::*luc* was upregulated, whereas expression of P*alsT*::*luc* was downregulated in a *tnrA* mutant strain (Fig.1 A and B).

We further tested the expression regulation of our nine candidate genes in the same conditions. Transcription rates from P*yuiA* and P*yvgT* were, respectively, three and fourfold increased in a *tnrA* mutant compared to wild type (Fig. S2). In contrast, the transcription rate of *luc* fused to P*appD*, P*dtpT*, P*pucR*, P*yrbD*, and P*ysnD* exhibited 3- to 30-fold lower levels in Δ*tnrA* cells (Fig. S2). These results validated the TnrA-dependent regulation of these seven genes. Regulation of *pgsB* could not be tested as this operon is not expressed in the strain 168 lineage (Do et al. 2011). The P*ydaB* fusion was not regulated in response to *tnrA* deletion in the conditions used (data not shown). This result suggested that other transcription factors could be involved in this regulation masking TnrA activity or that the TnrA box identified is not properly positioned with respect to the promoter and thus not functional.

Eleven promoter-proximal TnrA-binding sites were identified and located in regions without a predicted TnrA box. We tested the ability of TnrA to mediate their regulation with transcriptional fusions between promoter regions and the *luc* reporter gene. P*yfiR* showed a slight 1.92-fold activation (±0.08) by TnrA while P*hom* and P*pycA* were 8 and 2-fold repressed by TnrA, respectively (Fig. S1, S2). These results demonstrated the TnrA-dependent regulation of these three transcription units. On the opposite, the expression from P*braB*, P*iscS* (divergently transcribed from P*brabB*), P*codV*, P*tdh*, P*yclM*, P*yclN* (divergently transcribed from P*yclM*), P*ykkC*, P*yflL*, P*ypqP*, and P*yugK* was not affected by the absence of TnrA in the conditions used (data not shown).

We observed a full overlap between the set of TnrA-binding sites detected in minimal medium with glutamate and in LB medium. Thus, we examined the regulation of the 20 newly identified promoter regions bound by TnrA in LB medium. No effect of *tnrA* deletion on the expression of those 20 promoters was observed in this medium, as expected (data not shown).

### Definition of a new TnrA box consensus and of the TnrA primary regulon

Subsequently, we used the MEME standard bioinformatic method (Bailey et al. 2006) to redefine the TnrA box consensus from the previously known directly regulated TnrA regulon and from the seven newly identified direct TnrA-regulated genes *yuiA*, *yvgT*, *appD*, *dtpT*, *pucR*, *yrbD*, and P*ysnD*. The alignment of all the TnrA boxes is shown in Figure S3. The resulting new TnrA box consensus sequence exhibited a dyad symmetry as previously found in Nakano et al. (1995) (Fig.3B). Remarkably, three highly conserved nucleotides were detected in mid-positions 8, 9, and 10.

We used this newly defined TnrA box consensus to search TnrA box motifs among the 17 TnrA-binding sites identified in ChIP-on-chip, which did not harbor a previously predicted TnrA box. In this analysis, we considered the genomic regions representing 100 bp centered at each TnrA-binding site. As a result, a TnrA box was detected in: (i) the promoter region of the TnrA-regulated genes *yfiR*, *hom*, and *pycA*; (ii) the promoter regions of *braB*, *ykkC*, *yflL*, *ypqP*, and *yugK*, whose expression was not affected in a *tnrA* mutant in the tested conditions; (iii) the intragenic binding sites of *kamA* and *ykoH* genes (Fig. S3).

Altogether, the results presented above allowed us to identify 10 new promoter regions harboring a TnrA box and directly regulated and bound by TnrA in vivo (Table2). The combination of our ChIP-on-chip results with data from previous studies (Yoshida et al. 2003) allowed us to define the TnrA primary regulon, which is composed of 35 promoter regions fulfilling three criteria: (i) TnrA binding in ChIP-on-chip experiments and/or in previous in vitro studies; (ii) presence of a TnrA box; and (iii) TnrA-dependent expression regulation.

**Table 2:** Identification of 10 additional promoter regions belonging to the TnrA primary regulon

Gene	Sequence of TnrA box^1^	TnrA-binding sites^2^	Expression ratio^3^
Genes positively regulated by TnrA			
* appD*	-119 TGTAATAATATACAACT	−95	0.13
* dtpT*	-212 TCTAAAATTTTATTAAA; -175 TGTAAGAAAATCTCACG	−271; −151	0.33
* pucR*	-140 TGTCAGTTTATGTAACA	−224	0.1
* yfiR*	-31 GGTAAGAAAATTGCAGA^*^	−51	0.5
* ysnD*	-141 TGGAAGATTTTATAACA; -97 TGACAGATCATCTTGCA	−142; −76	0.33
* yrbD*	-96 GGTCATATAATGTGACA	−121	0.04
Genes negatively regulated by TnrA			
* hom*	-203 GAGGAGAAAATCTGACT^*^	−55; −187	8
* pycA*	-87 TGTTTATCTGTAAAAAA^*^	−87	2
* yuiA*	-78 TGTCACATGATCTGACT	−77	3
* yvgT*	-39 CGTCAGAAAATTTAACA	+53	4

1 Positions of TnrA boxes are indicated relative to the translational start site (+1) according to (Yoshida et al. 2003) and the RegTransBase database (Cipriano et al. 2013). Asterisks indicate TnrA boxes predicted in this work from the newly defined TnrA box consensus.

2 Positions of in vivo TnrA-binding sites correspond to the top of each peak detected by ChIP-on-chip and are indicated relative to the translational start site (+1).

3 The ratio of expression for each gene (Δ*tnrA* mutant/wild-type) was calculated as the average of three independent experiments obtained with the *luc* transcriptional fusions.

Subsequently, we verified if a different consensus could be detected if the 42 sites were considered using the MEME method. We did not impose a constraint that the motif must be an inverted repeat sequence on the search. When all the sites were used, no difference could be detected in the TnrA box consensus. Moreover, if only the promoters not regulated by TnrA were considered, no consensus at all was found. This suggested that TnrA probably does not directly interact on these sites.

### Identification of additional TnrA-regulated genes associated with a TnrA box

In order to obtain a full extent of the TnrA regulon, we also examined the regulation of genes associated with a predicted TnrA box (Cipriano et al. 2013), even without the detection of in vivo TnrA-binding sites. Four genes were concerned: *pucA*, *pucI*, *ycdE*, and *yumC* (Table S1). Expression of the corresponding promoter regions was compared in wild-type and Δ*tnrA* genetic backgrounds. No effect of TnrA could be detected for the P*ycdE* expression in the conditions used (data not shown). Expression from P*pucI* and P*yumC* was 3-fold upregulated in a *tnrA* mutant, whereas the expression from P*pucA* was fully downregulated in Δ*tnrA* cells (Figs. S1 and S2). Thus, we identified a second set of three TnrA-dependent transcription units encoding seven genes, *pucABCDE*, *pucI*, and *yumC*.

### Genes under the control of TnrA respond differently to the presence of glutamine

GS enzyme acts as a glutamine sensor, transducting the glutamine signal to TnrA. In the GS sequestered state, TnrA cannot control its regulon (Wray et al. 2001). We examined the expression of the 13 newly identified TnrA-regulated promoter regions in minimal medium with both glutamate and glutamine as nitrogen sources, during exponential phase (Fig. S1 and S2). The seven promoters positively regulated by TnrA were downregulated in the presence of glutamine, as expected. In contrast, addition of glutamine enhanced the expression of 5 TnrA-repressed genes. The same expression profiles were observed in Δ*tnrA* and wild-type strains (Fig. S1 and S2). These results confirmed that TnrA is the major regulator to mediate regulation of these genes in response to glutamine availability.

Regulation of the *hom* gene in response to glutamine presented an unexpected profile. Expression of this gene remained at low level in the wild-type as well as in Δ*tnrA* cells in this condition (Fig. S1). This was in agreement with a previous report, which showed by transcriptomic analysis that *hom* expression was downregulated by glutamine (Ye et al. 2009).

## Discussion

### ChIP-on-chip data allowed the TnrA primary regulon definition

Among the 42 TnrA-binding sites identified by ChIP-on-chip in this study, 16 belong to the known TnrA regulon (Fig.3A) (Yoshida et al. 2003). As some regions are involved in the regulation of two divergent promoters, in total we recovered 19 out of 25 well-characterized TnrA-regulated promoters. We also confirmed that TnrA directly regulates the *yrbD* and *ysnD* genes (Fig. S1 and S2) (Yoshida et al. 2003; Tojo et al. 2004). Remarkably, TnrA-binding sites detected in vivo were centered on the TnrA boxes delimited in previous gel mobility shift assays (Yoshida et al. 2003) (Table S2). In addition, the detection of two TnrA-enriched regions in front of the *dtpT*, *glnR*, *ycsF*, and *ysnD* genes correlates with the presence of two TnrA box motifs predicted in silico (Table S1). Altogether, our study illustrates the power of ChIP-on-chip approaches, generating pertinent data to finely delineate a transcriptional regulator in vivo binding sites.

ChIP-on-chip data combined with real-time in vivo transcriptional profiling enabled us to validate the functionality of seven in silico predicted TnrA boxes (Fig.2). Moreover, we identified three TnrA-dependent promoter regions, which match with the newly defined TnrA box consensus. Altogether, the ChIP-on-chip approach allowed us to define the TnrA primary regulon, which is now composed of 35 transcriptional units among which 10 were newly identified in this work (Table2). The microarray results previously published (Yoshida et al. 2003) were obtained from RNA samples prepared from cells harvested in middle logarithmic growth. In this work, we analyzed candidate genes expression, live during growth, using in vivo transcriptional profiling. We can see in Figure S1 and S2 that the maximal TnrA-dependent regulatory effect was observed within a 30-min window. Variations between expression profiles could explain why some newly identified TnrA-dependent genes in our work have not been detected in the previous transcriptomic study.

The TnrA primary regulon delimitation finally allowed us to refine the TnrA box consensus (Fig.3B). A sequence analysis of these 35 promoters using the MEME suite produced a consensus not only composed of an inverted repeat sequence (Nakano et al. 1995) but also of three highly conserved nucleotides at the mid-positions 8, 9, and 10 (Fig.3B). Subsequently, the improvement of the TnrA box consensus enabled the identification of new TnrA boxes present in the proximity of in vivo TnrA-binding sites (Fig. S3). Thus, the ChIP-on-chip methodology is also a powerful strategy to identify regulatory sites that have escaped previous in silico screenings.

### TnrA might regulate other genes expression in different growth conditions

Our work also led to the identification of a TnrA secondary regulon, whose members do not fulfill all the criteria presented above. By ChIP-on-chip, we identified 10 sites in promoter regions that were not differentially regulated in a Δ*tnrA* strain according to the transcriptional fusions analysis. These unexpected protein–DNA interactions that could not be identified using transcriptional profiling have been observed before (Blanka et al. 2014). Remarkably, the TnrA-binding sites detected in LB and in minimal medium with glutamate fully overlapped while TnrA did not play any significant regulatory role in LB medium. The cross-linking agent used in the Chip-on-chip procedure could reveal transient interactions between TnrA and the chromosome that would not result in transcriptional regulation. In addition, the binding and dissociation properties of the TnrA protein on DNA may change depending on the conditions of growth. We postulate that TnrA DNA-binding affinity could be weaker in LB than in minimal medium with glutamate, allowing TnrA to transiently bind on its specific targets but preventing TnrA to play its regulatory role. Hence, the ChIP-on-chip approach could give access to all the targets of a given transcriptional regulator even if some of the targets are not functional in the conditions used. In this set of 10 TnrA-promoter-proximal binding sites, *ydaB* and *pgsB* harbor a predicted TnrA box (Cipriano et al. 2013). Moreover, the new TnrA box consensus allowed the detection of a TnrA box in the promoter regions of *braB*, *ykkC*, *yflL*, *ypqP*, and *yugK* (Fig. S3). As we did not observe a TnrA-dependent regulation of these genes in the conditions used, we propose that TnrA might also play a regulatory role in specific unknown conditions. It was recently shown that the CodY regulator also binds upstream of the *braB* and *ypqP* genes (Belitsky and Sonenshein 2013), which may account for the absence of Δ*tnrA* effect on their expression.

The identification of six intragenic TnrA-binding sites was also intriguing since, to our knowledge, no TnrA intragenic target has ever been described. In silico analysis also showed the presence of a TnrA box match based on our new TnrA box consensus inside the *kamA* and *ykoH* genes (Table S2). It is possible that TnrA could bind to these intragenic sites to mediate repression by a roadblock mechanism, as described for the *B. subtilis* CcpA and CodY regulators (Choi and Saier 2005; Belitsky and Sonenshein 2011).

In vivo TnrA-binding sites that did not harbor a potential TnrA box suggest that TnrA could recognize sequences with lower motif similarity, different consensus sequences or that other factors could be involved in the DNA binding. Thus, our results highlight a secondary TnrA regulon, which is composed of 16 genomic regions, bound by TnrA in vivo, for which the conditions of a potential TnrA-dependent regulation remain still unknown (Fig.3A) (Table S2). As the disruption of nitrogen metabolism in Δ*tnrA* cells affects many cellular processes, the direct role of TnrA binding to promoter regions could be masked by other regulatory effects. Finally, we identified a TnrA tertiary regulon, which is composed of promoter regions whose expression is altered in a *tnrA* mutant but without experimental evidence of direct in vivo or in vitro TnrA binding (Table S2). This regulon includes *pucA*, *pucI*, and *yumC*, which exhibit a predicted TnrA box as well as *citB* (Blencke et al. 2006), *guaD* (Nygaard et al. 2000) and the 19 TnrA-regulated promoters associated with a putative TnrA box without in vitro binding (Yoshida et al. 2003).

Altogether, this study allowed us to validate three groups in the TnrA regulon. However, the composition of the secondary and tertiary regulons cannot be clearly delimited and is opened to permutations with the primary regulon depending on the discovery of yet unknown conditions involving TnrA-dependent regulation.

### TnrA links nitrogen metabolism to other cellular processes

Thirteen additional targets are now part of the TnrA regulon (10 in the primary and 3 in the secondary regulon). It is worthwhile mentioning that several of the positively regulated TnrA genes are involved in the transport of peptides (*dtpT*), oligopeptides (*appDABC*), and amino acids (*yrbD*) (Michna et al. 2014). Our data support the physiological role of TnrA in controlling the uptake of alternative substrates as nitrogen source, as already mentioned in Yoshida et al. (2003). In addition, we detected TnrA-binding in the *ywzF* promoter region, which is located upstream of the *ureABC* operon, involved in urea assimilation as an alternative nitrogen source. Expression of *ureABC* is driven from a P3 promoter located 839-bp upstream of the *ureA* start codon (Wray et al. 1997). The *ure*P3 promoter overlaps with the *ywzF* promoter region. The *ure*P3 promoter region contains two predicted TnrA-binding sites (Brandenburg et al. 2002). Although the *ureABC* operon was shown to be induced by TnrA, it was reported that its transcription is indirectly depended on TnrA (Brandenburg et al. 2002; Yoshida et al. 2003). According to our data, the *ywzF* DNA region containing the *ure*P3 promoter may be directly involved in TnrA-dependent activation of *ureABC*.

Some of the genes negatively regulated by TnrA are linked to the amino acids metabolism. *pycA* encodes the pyruvate carboxylase involved in the oxaloacetate pool replenishment. In addition to initiating the Krebs cycle oxaloacetate can be converted to aspartate, the threonine precursor. The *hom* gene encodes the homoserine dehydrogenase involved in the biosynthesis of threonine, the isoleucine precursor. TnrA is already known to repress the *ilv-leu* operon (Tojo et al. 2004). The control of the *pycA* and *homthrCB* operons by TnrA is a novel regulatory link between the nitrogen metabolism and amino acid metabolism.

We also showed that TnrA fully contributes to the control of purine utilization. TnrA directly represses *pucI* and also activates *pucA* and *pucR* expression. The PucR regulator induces *pucJKLM* expression in nitrogen-limited conditions (Beier et al. 2002). Therefore, the *pucR* TnrA-dependent upregulation can explain the indirect positive regulation of the *pucJKLM* operon by TnrA (Yoshida et al. 2003). This is an obvious interplay between nitrogen assimilation and purine catabolism regulatory networks.

Finally, our study highlights the TnrA-dependent regulation of genes connected to the oxidative stress response. In previous transcriptomic study, the *yuiA* gene has been shown to be derepressed in a *perR* mutant lacking the major regulator for oxidative stress response (Helmann et al. 2003). The function of the YuiA protein remains still unknown. According to the classification of *B. subtilis* promoter regions, the *yuiA* gene is co-regulated with *ilvE*, a branched-chain amino acid aminotransferase, and *ctrA*, a branched-chain amino acid transporter (cluster A142) (Nicolas et al. 2012). Intracellular submicromolar concentrations of hydrogen peroxide are sufficient to disrupt metabolism by damaging iron–sulfur enzymes (Jang and Imlay 2007), as, for example, the *Escherichia coli* dehydratase involved in the biosynthesis of leucine (Jang and Imlay 2007). Therefore, the YuiA protein could play a role in nitrogen-limiting conditions at the interface between the amino acid metabolism and the response to oxidative stress. We also identified the TnrA-repressed gene *yumC*. It encodes a ferredoxin-NAD(P)+ oxidoreductase, which can play a role in protecting cells against oxidative stress (Bianchi et al. 1995; Pomposiello and Demple 2000; Lee et al. 2006). As the non-enzymatic antioxidant molecule NADPH helps to maintain an intracellular reducing environment, the YumC enzyme could be involved in maintaining a strong reducing environment in cells growing in nitrogen-limiting conditions. Overall, our data underline a potential link between nitrogen availability and oxidative stress response. Further investigations are required to define the exact role of the *yuiAB* and *yumC* genes in the nitrogen metabolism and/or the oxidative stress response.

## Supporting Information

**Figure S1.** Direct involvement of TnrA as repressor in the control of *yuiA*, *yvgT*, *hom*, *pycA*, *pucI*, and *yumC* expression. Strains were cultivated in minimal medium with glutamate (left panels A, C, E, G, I, and K) or with glutamate + glutamine (right panels B, D, F, H, J, and L) as nitrogen source. Growth (OD_600nm_) was monitored every 5 min: blue circles, wild-type; red circles, Δ*tnrA*. Promoter activity (RLU/OD) of each transcriptional fusion with the *luc* reporter gene is indicated: blue lines, in the wild-type; red lines, in Δ*tnrA* cells. Growth and promoter activity were followed for the six genes *yuiA*, *yvgT*, *hom*, *pycA*, *pucI*, and *yumC*. For each strain, one representative curve, out of three independent replicates realized, is shown. Note that the X-axes are different for paired panels to get a better view of the data. **Figure S2.** Direct involvement of TnrA as activator in the control of *appD*, *dtpT*, *yrbD*, *pucR*, *ysnD*, *yfiR*, and *pucA* expression. Strains were cultivated in minimal medium with glutamate (left panels A, C, E, G, I, K, and M) or with glutamate + glutamine (right panels B, D, F, H, J, L, and N) as nitrogen source. Growth (OD_600nm_) was monitored every 5 min: blue circles, wild-type; green circles, Δ*tnrA*. Promoter activity (RLU/OD) of each transcriptional fusion with the *luc* reporter gene is indicated: blue lines, in the wild type; green lines, in Δ*tnrA* cells. Growth and promoter activity were followed for the seven genes *appD*, *dtpT*, *yrbD*, *pucR*, *ysnD*, *yfiR*, and *pucA*. For each strain, one representative curve, out of three independent replicates realized, is shown. Note that the Y-axes are different for some paired panels to get a better view of the data. **Figure S3.** Alignment of TnrA box sequences from the TnrA primary and secondary regulon.

Table S1 Mapping of TnrASPA DNA-binding sites by ChIP-on-chip.

Table S2 Classification of TnrA-dependent transcription units in primary, secondary, and tertiary regulons.

Table S3 Oligonucleotide primers used in this study.
