# Supplementary material for: Genome-wide mapping of TnrA-binding sites provides new insights into the TnrA regulon in Bacillus subtilis
Source: Microbiologyopen. 2015 Mar 8;4(3):423–35. doi: 10.1002/mbo3.249 (PMC4475385; doi:10.1002/mbo3.249)

Supplemental text for

Genome-wide mapping of TnrA-binding sites provides new insights into the TnrA regulon in *Bacillus subtilis*

Nicolas Mirouze [a,b], Elena Bidnenko [a,b], Philippe Noirot [a,b], Sandrine Auger[a,b,1]

[a] INRA, UMR1319 Micalis, F-78352 Jouy-en-Josas, France

[b]AgroParisTech, UMR Micalis, F-78352 Jouy-en-Josas, France

[1]Corresponding author: Sandrine Auger, Email: sandrine.auger@jouy.inra.fr.

Supplemental Figures Legend.

Figure S1. Direct involvement of TnrA as repressor in the control of *yuiA*, *yvgT*, *hom*, *pycA*, *pucI* and *yumC* expression.

Strains were cultivated in minimal medium with glutamate (left panels A, C, E, G, I and K) or with glutamate + glutamine (right panels B, D, F, H, J and L) as nitrogen source. Growth (OD_{600nm}) was monitored every 5 min: blue circles, wild-type; red circles, Δ*tnrA*. Promoter activity (RLU/OD) of each transcriptional fusion with the *luc* reporter gene is indicated: blue lines, in the wild-type; red lines, in Δ*tnrA* cells. Growth and promoter activity were followed for the 6 genes *yuiA*, *yvgT*, *hom*, *pycA*, *pucI* and *yumC*. For each strain, one representative curve, out of three independent replicates realized, is shown. Note that the X-axes are different for paired panels to get a better view of the data.

Figure S2. Direct involvement of TnrA as activator in the control of *appD*, *dtpT*, *yrbD*, *pucR*, *ysnD*, *yfiR* and *pucA* expression.

Strains were cultivated in minimal medium with glutamate (left panels A, C, E, G, I, K and M) or with glutamate + glutamine (right panels B, D, F, H, J, L and N) as nitrogen source. Growth (OD_{600nm}) was monitored every 5 min: blue circles, wild-type; green circles, Δ*tnrA.* Promoter activity (RLU/OD) of each transcriptional fusion with the *luc* reporter gene is indicated: blue lines, in the wild type; green lines, in Δ*tnrA* cells. Growth and promoter activity were followed for the 7 genes *appD*, *dtpT*, *yrbD*, *pucR*, *ysnD*, *yfiR* and *pucA*. For each strain, one representative curve, out of three independent replicates realized, is shown. Note that the Y-axes are different for some paired panels to get a better view of the data.

Figure S3. Alignment of TnrA box sequences from the TnrA primary and secondary regulon.

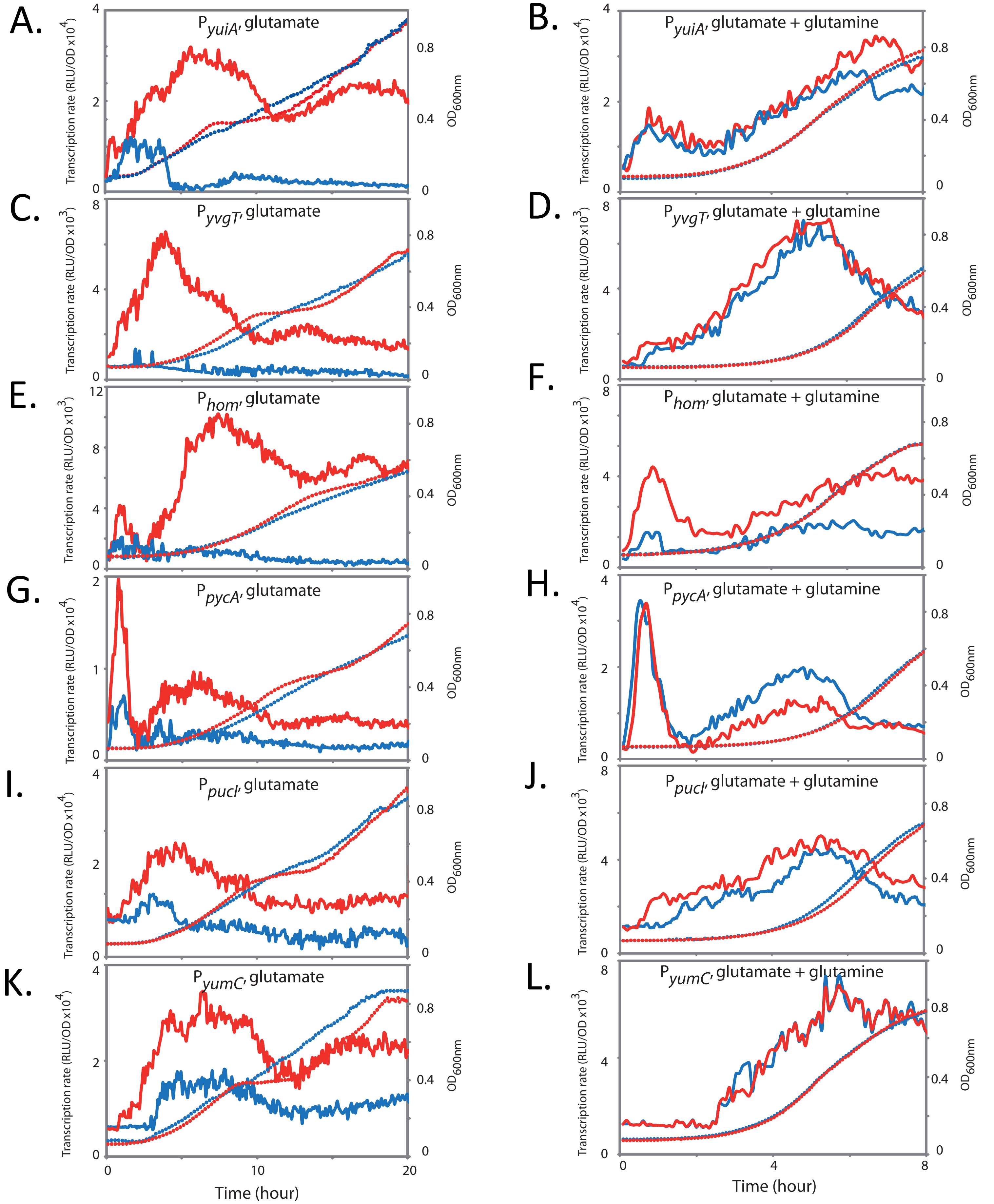
A.
P yuiA' glutamate
B.
P yuiA' glutamate + glutamine
C.
P yvgT' glutamate
D.
P yvgT' glutamate + glutamine
E.
P hom' glutamate
F.
P hom' glutamate + glutamine
G.
P pycA' glutamate
H.
P pycA' glutamate + glutamine
I.
P pucI' glutamate
J.
P pucI' glutamate + glutamine
K.
P yumC' glutamate
L.
P yumC' glutamate + glutamine
Transcription rate (RLU/OD x10^4)
Transcription rate (RLU/OD x10^3)
OD600nm
Time (hour)

Mirouze et al., Figure S2

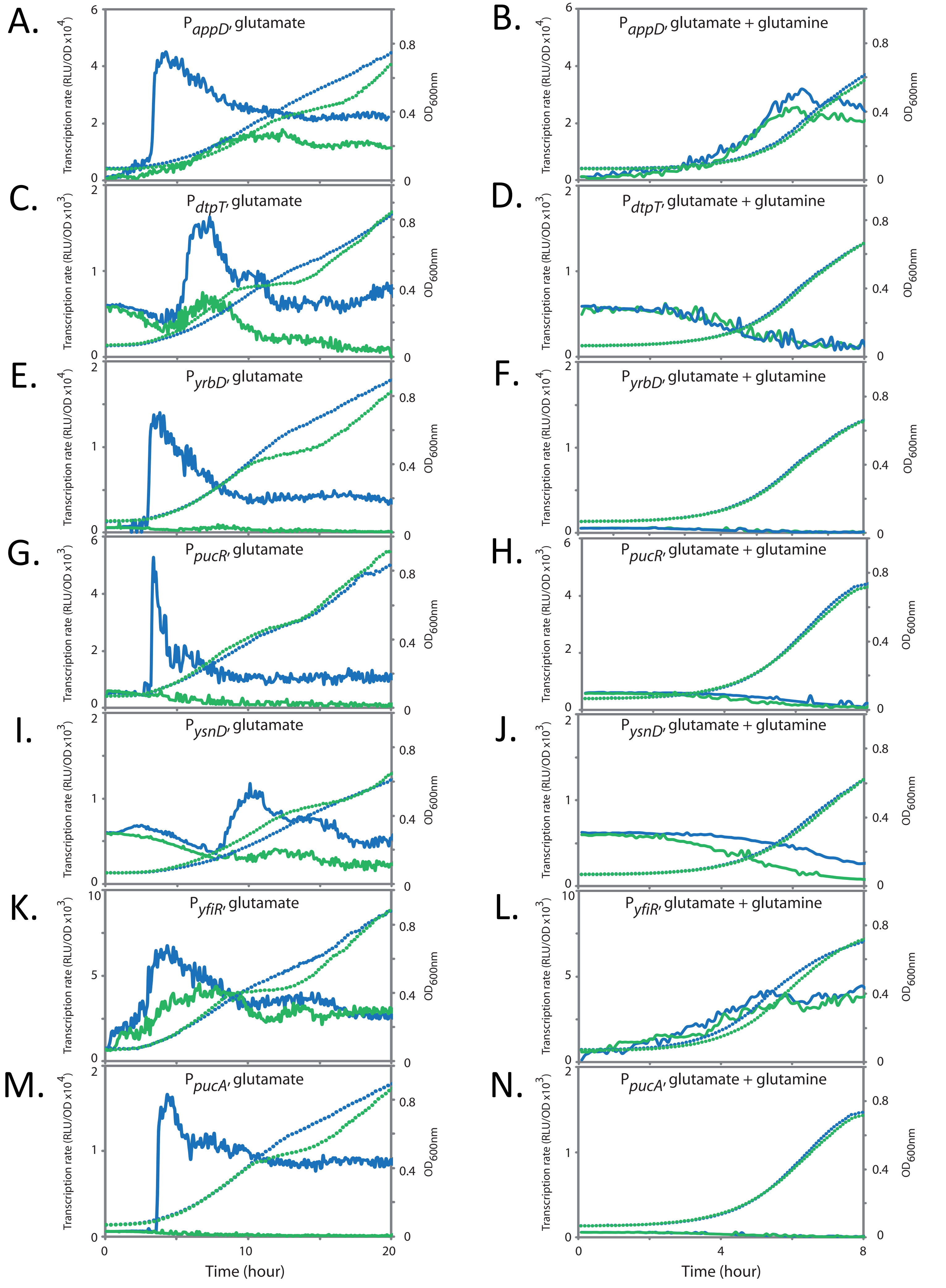

Mirouze et al., Figure S3

glnQ	(+)	-28	GGTCAAATTTTCTGATA
nasA	(-)	-82	TGTAAGTTTTTGTGACA
nasD	(+)	-85	TGTTACATTTTATAACA
nrgA	(-)	-102	TGTAAGATTTCCTGACA
oppA	(-)	-231	CGTTAGTTTTTCTTCCA
pucJ	(+)	-140	TGTTACATTTTCTTACA
tnrA	(+)	-124	TGTTAATTTTTCTTCCG
ykzB	(-)	-87	TGTTAATTTTTCTTCCG
ureA	(-)	-938	TGTTATATTTATTCACA
ycsF	(+)	-145	TGGTAGGTTTTCTGACA
ycsF	(-)	-52	TGTCATTTATTGTCCCG
gabP	(+)	-103	TGGTATATTTTCTTACA
yrbD	(-)	-96	TGTCACATTATATGACC
appD	(-)	-119	AGTTGTATATTATTACA
dtpT	(+)	-212	TCTAAAATTTTATTAAA
dtpT	(-)	-175	CGTGAGATTTTCTTACA
pucR	(+)	-140	TGTCAGTTTATGTAACA
ysnD	(+)	-141	TGGAAGATTTTATAACA
ysnD	(+)	-97	TGACAGATCATCTTGCA
ywrD	(+)	-83	CGTCACTTTTTCTGCCG
glnR	(+)	-82	TGTTAAGAATCCTTACA
glnR	(-)	-59	TGTTATATTATGTGTCA
gltA	(+)	-51	TGTTAGATTTTATGACC
ilvB	(-)	-736	TGACAGATCATCTTGCA
ilvB	(-)	-692	TGGAAGATTTTATAACA
pel	(+)	-27	TGTGAGATTTTATTCCA
yoyD	(+)	-61	TGTGATCTTTTCTTACA
ywdI	(-)	-92	TGTTATTTTTTCTGACA
yycC	(+)	-66	TGTGACATCTTCTTACA
yttA	(-)	-69	TGTTAGATTTTTTCACG
ywlF	(-)	-154	TTGCAGATTTTCTGACA
alsT	(-)	-61	TGTTAGATTTTTTAACA
degU	(-)	-159	TGTCATCGTTCCTTCCA
yuiA	(-)	-78	AGTCAGATCATGTGACA
yvgT	(-)	-39	TGTTAAATTTTCTGACG
yfiR	(-)	-31	TCTGCAATTTTCTTACC
hom	(-)	-203	AGTCAGATTTTCTCCTC
pycA	(+)	-87	TGTTTATCTGTAAAAAA

TnrA primary regulon

braB	(-)	-55	TGTTAGATTTTCTTCCT
ydaB	(-)	-55	TGTTAGATTTTCTTCCT
yflL	(+)	-345	CGTCAGATTTTCTCCAA
ykkC	(-)	-243	TGTAAGATTTTCTTTCA
ypqP	(-)	+20	AAACAGCTTTTCAGATA
yugK	(-)	-45	AAACAGATTTTCCATTG
kamA	(+)	+168	CGTCAGAATTTCTACCA
ykoH	(-)	+330	CGTCAGGTTTCCTCACA

TnrA secondary regulon

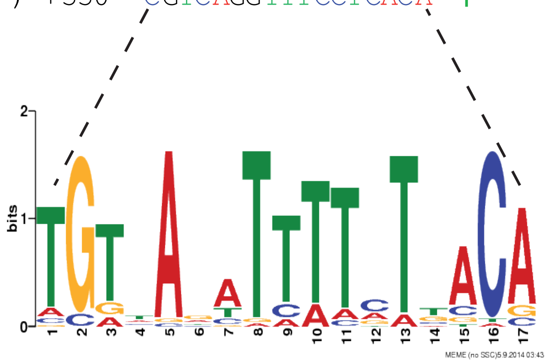

Supplement: Supplementary file 1 — Figure S1. Direct involvement of TnrA as repressor in the control of yuiA, yvgT, hom, pycA, pucI, and yumC expression. Strains were cultivated in minimal medium with glutamate (left panels A, C, E, G, I, and K) or with glutamate + glutamine (right panels B, D, F, H, J, and L) as nitrogen source. Growth (OD600nm) was monitored every 5 min: blue circles, wild-type; red circles, ΔtnrA. Promoter activity (RLU/OD) of each transcriptional fusion with the luc reporter gene is indicated: blue lines, in the wild-type; red lines, in ΔtnrA cells. Growth and promoter activity were followed for the six genes yuiA, yvgT, hom, pycA, pucI, and yumC. For each strain, one representative curve, out of three independent replicates realized, is shown. Note that the X-axes are different for paired panels to get a better view of the data. Figure S2. Direct involvement of TnrA as activator in the control of appD, dtpT, yrbD, pucR, ysnD, yfiR, and pucA expression. Strains were cultivated in minimal medium with glutamate (left panels A, C, E, G, I, K, and M) or with glutamate + glutamine (right panels B, D, F, H, J, L, and N) as nitrogen source. Growth (OD600nm) was monitored every 5 min: blue circles, wild-type; green circles, ΔtnrA. Promoter activity (RLU/OD) of each transcriptional fusion with the luc reporter gene is indicated: blue lines, in the wild type; green lines, in ΔtnrA cells. Growth and promoter activity were followed for the seven genes appD, dtpT, yrbD, pucR, ysnD, yfiR, and pucA. For each strain, one representative curve, out of three independent replicates realized, is shown. Note that the Y-axes are different for some paired panels to get a better view of the data. Figure S3. Alignment of TnrA box sequences from the TnrA primary and secondary regulon. [file mbo30004-0423-sd1.pdf]
