# Supplementary material for: Genome-wide mapping of TnrA-binding sites provides new insights into the TnrA regulon in Bacillus subtilis
Source: Microbiologyopen. 2015 Mar 8;4(3):423–35. doi: 10.1002/mbo3.249 (PMC4475385; doi:10.1002/mbo3.249)
Supplement: Table S3 — Oligonucleotide primers used in this study. [file mbo30004-0423-sd4.doc]

**TABLE S3 Oligonucleotide primers used in this study**

| **Name** | **Sequence (5'→3')** |
| --- | --- |
| F- pUC18-luc | CTCTAGAGGATCCCCGGGTACCAG |
| R- pUC18-luc | TCGACCTGCAGGCATGCAAGCTTG |
| F-nasB | **CAAGCTTGCATGCCTGCAGGTCGA**CCGCCGACACGATCTGAAATC |
| R-nasB | **CTGGTACCCGGGGATCCTCTAGAG**GGCGAAGCTCGTAAGTTTTCTCC |
| F-hom | **CAAGCTTGCATGCCTGCAGGTCGA**GTGAACTGACATTTGAACATGAGG |
| R-hom | **CTGGTACCCGGGGATCCTCTAGAG**GATTGTCCTCTTGGAAAAGACAAT |
| F-yuiA | **CAAGCTTGCATGCCTGCAGGTCGA**CGTCGCGCCTGCTGCGGGAAC |
| R-yuiA | **CTGGTACCCGGGGATCCTCTAGAG**CTGATAATATACCATATCAAGC |
| F-yfiR | **CAAGCTTGCATGCCTGCAGGTCGA**CTGAAATCGGCCGTGCCCTC |
| R-yfiR | **CTGGTACCCGGGGATCCTCTAGAG**GCTTTCTGCAATTTTCTTACCTATAAGTG |
| F-pycA | **CAAGCTTGCATGCCTGCAGGTCGA**GGGCATGTTGCCGGAAACGCGC |
| R-pycA | **CTGGTACCCGGGGATCCTCTAGAG**CTTACCCGAATGGAATATAATC |
| F-appD | **CAAGCTTGCATGCCTGCAGGTCGA**GGATTAAAAGAGGAGGCGATC |
| R-appD | **CTGGTACCCGGGGATCCTCTAGAG**CCCTCTTGTTGTCAAACGTTTTC |
| F-tdh | **CAAGCTTGCATGCCTGCAGGTCGA**CGTGCAAACA GCGGATAACCGC |
| R-tdh | **CTGGTACCCGGGGATCCTCTAGAG**CTTGTTATATTCGGATTGTACCAT |
| F-pucR | **CAAGCTTGCATGCCTGCAGGTCGA**GCGGACATGTTTCAACCGAAAC |
| R-pucR | **CTGGTACCCGGGGATCCTCTAGAG**CATTGGTTAAGGTGAATAATTTTC |
| F-dtpT | **CAAGCTTGCATGCCTGCAGGTCGA**CGTCACCTTCGCCATATCCTTC |
| R-dtpT | **CTGGTACCCGGGGATCCTCTAGAG**CATATTCTTATATAATACTTGTGTC |
| F-yrbD | **CAAGCTTGCATGCCTGCAGGTCGA**CCGCATATCA TGCACTCTACAA |
| R-yrbD | **CTGGTACCCGGGGATCCTCTAGAG**CCTCACTTCAAATTCGAATACATG |
| F-ysnD | **CAAGCTTGCATGCCTGCAGGTCGA**CGGGGAAGGA TATGTACCAACC |
| R-ysnD | **CTGGTACCCGGGGATCCTCTAGAG**GCAAGATATCATTAATGTATGCCG |
| F-yvgT | **CAAGCTTGCATGCCTGCAGGTCGA**CGTGAA CGTCATGTTC CATGG |
| R-yvgT | **CTGGTACCCGGGGATCCTCTAGAG**CCCTTTTATGTTTGTTAAATTTTC |
| F-alsT | **CAAGCTTGCATGCCTGCAGGTCGA**TGGTTGGTGAATAGATCTGTTAC |
| R-alsT | **CTGGTACCCGGGGATCCTCTAGAG**CATCAATTCAATACGAATTCGATT |
| F-yclN | **CAAGCTTGCATGCCTGCAGGTCGA**CAGAACCGCTCC GTGAGAATGTG |
| R-yclN | **CTGGTACCCGGGGATCCTCTAGAG**GAACGTATCTCCTACTCCGCAAG |
| F-pucI | **CAAGCTTGCATGCCTGCAGGTCG**AGTATATGTTC CCGAAAGATCAG |
| R-pucI | **CTGGTACCCGGGGATCCTCTAGAG**CCTTTCCATATTGTCTATATGTCG |
| F-pucA | **CAAGCTTGCATGCCTGCAGGTC**GAAGCCTGCCGC ACCGAGGATGCA |
| R-pucA | **CTGGTACCCGGGGATCCTCTAGAG**TCCTCTCGAAAATTGCCGTGAG |
